# Outbreak of severe community-acquired bacterial infections among children in North Rhine-Westphalia (Germany), October to December 2022

**DOI:** 10.1007/s15010-023-02165-x

**Published:** 2024-02-16

**Authors:** Sarah C. Goretzki, Mark van der Linden, Andreas Itzek, Tom Hühne, Roland O. Adelmann, Firas Ala Eldin, Mohamed Albarouni, Jan-Claudius Becker, Martin A. Berghäuser, Thomas Boesing, Michael Boeswald, Milian Brasche, Francisco Brevis Nuñez, Rokya Camara, Clara Deibert, Frank Dohle, Jörg Dolgner, Jan Dziobaka, Frank Eifinger, Natalie Elting, Matthias Endmann, Guido Engelmann, Holger Frenzke, Monika Gappa, Bahman Gharavi, Christine Goletz, Eva Hahn, Yvonne Heidenreich, Konrad Heimann, Kai O. Hensel, Hans-Georg Hoffmann, Marc Hoppenz, Gerd Horneff, Helene Klassen, Cordula Koerner-Rettberg, Alfred Längler, Pascal Lenz, Klaus Lohmeier, Andreas Müller, Frank Niemann, Michael Paulussen, Falk Pentek, Ruy Perez, Markus Pingel, Philip Repges, Tobias Rothoeft, Jochen Rübo, Herbert Schade, Robert Schmitz, Peter Schonhoff, Jan N. Schwade, Tobias Schwarz, Peter Seiffert, Georg Selzer, Uwe Spille, Carsten Thiel, Ansgar Thimm, Bartholomäus Urgatz, Alijda van den Heuvel, Tan van Hop, Verena Giesen, Stefan Wirth, Thomas Wollbrink, Daniel Wüller, Ursula Felderhoff-Müser, Christian Dohna-Schwake, Thiên-Trí Lâm, Heike Claus, Nora Bruns

**Affiliations:** 1 Department of Pediatrics I (Neonatology, Pediatric Intensive Care, Pediatric Neurology, and Pediatric Infectious Diseases), University Hospital Essen, University of Duisburg-Essen, Essen, Germany; 2 German Reference Laboratory for Streptococci, Department of Medical Microbiology, University Hospital RWTH Aachen, Aachen, Germany; 3 Department of General Pediatrics, Klinikum Oberberg, Kreiskrankenhaus Gummersbach, Gummersbach, Germany; 4 Department of General Pediatrics, Helios Hospital Schwelm, Schwelm, Germany; 5 Department of General Pediatrics, Marien-Hospital Gelsenkirchen, Gelsenkirchen, Germany; 6 Department of Pediatrics, Agaplesion Hospital Hagen, Hagen, Germany; 7 Division of Pediatric Intensive Care, Department of Pediatrics, Florence Nightingale Hospital Kaiserswerth, Düsseldorf, Germany; 8 Division of Pediatric Intensive Care, Department of Pediatrics, Protestant Hospital Bethel, University of Bielefeld, Bielefeld, Germany; 9 Department of Pediatrics, Sankt Franziskus Hospital Münster, Münster, Germany; 10 Division of Neonatology, Department of Pediatrics, University Hospital, RWTH University of Aachen, Aachen, Germany; 11 Division of Pediatric Intensive Care, Department of Pediatrics, Sana Hospitals Duisburg, Duisburg, Germany; 12 Division of Neonatology, Department of Pediatrics and Adolescent Medicine, GFO Hospital Bonn, Bonn, Germany; 13 Department of General Pediatrics, DRK Hospital Kirchen, Kirchen, Germany; 14 Department of Pediatrics, Pediatric Intensive Care Medicine, St. Vinzenz Hospital Paderborn, Paderborn, Germany; 15 Department of General Pediatrics, GFO Hospital Dinslaken, Dinslaken, Germany; 16 Department of Microbiology, University Hospital Essen, University of Duisburg-Essen, Essen, Germany; 17 Division of Pediatric Intensive Care, Department of Pediatrics, University Hospital, University of Cologne, Cologne, Germany; 18 Department of General Pediatrics, Evangelical Hospital Oberhausen, Oberhausen, Germany; 19 Department of General Pediatrics, St. Franziskus-Hospital Ahlen, Ahlen, Germany; 20 Department of General Pediatrics, Lukas-Hospital Neuss, Neuss, Germany; 21 Department of General Pediatrics, Märkisch Hospital Lüdenscheid, Lüdenscheid, Germany; 22 Department of General Pediatrics, Evangelical Hospital Düsseldorf, Düsseldorf, Germany; 23 Department of General Pediatrics, Marien-Hospital Witten, Witten, Germany; 24 Department of General Pediatrics, Städtische Kliniken Mönchengladbach, Elisabeth-Hospital Rheydt, Mönchengladbach, Germany; 25 Department of Pediatrics and Adolescent Medicine, Sankt Agnes Hospital, Bocholt, Germany; 26 Department of General Pediatrics, Hospital Soest gGmbH, Soest, Germany; 27 Division of Pediatric Intensive Care, Department of Pediatrics, Helios University Hospital Wuppertal, Witten/Herdecke University, Wuppertal, Germany; 28 Department of General Pediatrics, Mathias-Stiftung, Rheine, Germany; 29 Division of Pediatric Intensive Care, Department of Pediatrics, Children’s Hospital, Amsterdamer Str., Cologne, Germany; 30 Department of Pediatrics, Asklepios Clinic Sankt Augustin GmbH, Sankt Augustin, Germany; 31 Department of Pediatrics and Adolescent Medicine, Hochsauerland Hospital, Arnsberg, Germany; 32 Department of General Pediatrics, Marien-Hospital gGmbH, Wesel, Germany; 33 Department of Pediatrics, Gemeinschaftskrankenhaus Herdecke, University of Witten/Herdecke, Herdecke, Germany; 34 Department of General Pediatrics, Hospital Leverkusen GmbH, Leverkusen, Germany; 35 Division of Neonatology and Pediatric Cardiology, Department of General Pediatrics, Heinrich Heine University, Düsseldorf, Germany; 36 Department of Neonatology and Pediatric Intensive Care Medicine, University of Bonn, Bonn, Germany; 37 Division of Oncology and Haematology, Department of General Pediatrics, Hospital of Children and Adolescents, University of Witten/Herdecke, Datteln, Germany; 38 Department of Pediatrics, Elisabeth-Hospital Essen, Essen, Germany; 39 Division of Pediatric Intensive Care, Department of Pediatrics, Helios Hospital Krefeld, Krefeld, Germany; 40 Department of General Pediatrics, DRK Hospital Siegen gGmbH, Siegen, Germany; 41 Department of General Pediatrics, Porz, Cologne, Germany; 42 Division of Neonatology and Pediatric Intensive Care, University Children’s Hospital, Ruhr University of Bochum, Bochum, Germany; 43 Department of General Pediatrics, St. Antonius Hospital Kleve, Kleve, Germany; 44 Department of General Pediatrics, Hospital Mechernich GmbH, Mechernich, Germany; 45 Department of Pediatrics, Helios Clinic Duisburg, Duisburg, Germany; 46 Department of Pediatrics, Clemenshospital Münster, Münster, Germany; 47 Department of General Pediatrics, Evangelical Hospital Lippstadt, Lippstadt, Germany; 48 Department of General Pediatrics, Municipal Hospital Solingen, Solingen, Germany; 49 Division of Neonatology and Pediatric Intensive Care, Evangelical Hospital Hamm, Hamm, Germany; 50 Department of General Pediatrics, Herford, Germany; 51 Department of Pediatrics, St.-Clemens-Hospital Geldern, Geldern, Germany; 52 Department of General Pediatrics, Sana-Hospital Remscheid, Remscheid, Germany; 53 Department of General Pediatrics, AKH Hospital Viersen, Viersen, Germany; 54 Division of Pediatric Intensive Care, Department of Pediatrics, University Hospital Münster, Münster, Germany; 55 Department of General Pediatrics, Hospital Oberhausen Sterkrade gGmbH, Oberhausen, Germany; 56 Department of General Pediatrics, Bethanien Hospital Moers, Moers, Germany; 57 Department of Pediatrics, Helios Medical Center Niederberg, Velbert, Germany; 58 Division of Pediatric Intensive Care, Department of Pediatrics, Bergmannsheil Pediatric Hospital Gelsenkirchen Buer, Gelsenkirchen, Germany; 59 Department of Pediatrics and Adolescent Medicine, Christophorus Hospital, Coesfeld, Germany; 60 German National Reference Laboratory for Meningococci and Haemophilus Influenzae, Institute for Hygiene and Microbiology, University of Würzburg, Würzburg, Germany

**Keywords:** Surveillance, Outbreak, Children, Bacterial infections, Community acquired infections, Germany

## Abstract

**Purpose:**

In late 2022, a surge of severe *S. pyogenes* infections was reported in several European countries. This study assessed hospitalizations and disease severity of community-acquired bacterial infections with *S. pyogenes, S. pneumoniae, N. meningitidis*, and *H. influenzae* among children in North Rhine-Westphalia (NRW), Germany, during the last quarter of 2022 compared to long-term incidences.

**Methods:**

Hospital cases due to bacterial infections between October and December 2022 were collected in a multicenter study (MC) from 59/62 (95%) children's hospitals in NRW and combined with surveillance data (2016–2023) from the national reference laboratories for streptococci, *N. meningitidis*, and *H. influenzae*. Overall and pathogen-specific incidence rates (IR) from January 2016 to March 2023 were estimated via capture–recapture analyses. Expected annual deaths from the studied pathogens were calculated from national death cause statistics.

**Results:**

In the MC study, 153 cases with high overall disease severity were reported with pneumonia being most common (59%, *n* = 91). IRs of bacterial infections declined at the beginning of the COVID-19 pandemic and massively surged to unprecedented levels in late 2022 and early 2023 (overall hospitalizations 3.5-fold), with *S. pyogenes* and *S. pneumoniae* as main drivers (18-fold and threefold). Observed deaths during the study period exceeded the expected number for the entire year in NRW by far (7 vs. 0.9).

**Discussion:**

The unprecedented peak of bacterial infections and deaths in late 2022 and early 2023 was caused mainly by *S. pyogenes* and *S. pneumoniae*. Improved precautionary measures are needed to attenuate future outbreaks.

**Supplementary Information:**

The online version contains supplementary material available at 10.1007/s15010-023-02165-x.

## Introduction

Since the outbreak of the COVID-19 pandemic in 2020, children’s and adolescents’ health has been challenged in numerous ways. Among others, a decline of bacterial infections and distorted periodicity of seasonal infection waves were observed across the world and affected all age groups [1–5]. An unintended result of reduced infections is the lack of natural immunity, especially in the youngest.

Beginning in late fall 2022, rising numbers of severe infections by group A streptococci were reported from Spain, France, Great Britain, and other countries [6–9]. At the same time, a massive wave of acute viral and bacterial infections caused a near-collapse of the German paediatric health care system including out- and inpatient sectors and overwhelming pediatric intensive care capacities [10–13]. Pediatric health care providers in Germany claimed this was an unprecedented public health emergency [10–12].

To verify the self-declared public health emergency from bacterial infections, we conducted this study. It was designed to assess hospitalizations due to community-acquired bacterial infections by *Streptococcus pyogenes* (*S. pyogenes*), *Streptococcus pneumoniae* (*S. pneumoniae*), *Neisseria meningitidis* (*N. meningitidis*), and *Haemophilus influenzae* (*H. influenzae*) in the last quarter of 2022 compared to seasonally expected infection waves since 2016.

## Methods

### Study design

This is a retrospective multicenter study (MC) on community-acquired bacterial infections requiring hospitalization in children and adolescents in North Rhine-Westphalia/Germany (NRW) between October 1st and December 31st of 2022. The results were combined with four data sources to deduce long-term trends.

(1) For the MC study, clinical data were collected from 59 of 62 (95%) children’s hospitals in NRW. (2) Surveillance data from the German reference laboratory for streptococci (GRLS) and the national reference laboratory for *N. meningitidis* and *H. influenzae* (NRLMHi) were matched with cases from the MC study for capture–recapture (CRC) analyses. (3) Monthly reference laboratory-reported incidences from January 2016 to March 2023 were combined with population statistics. (4) The expected number of deaths from bacterial infections per year in children < 15 years was calculated from national death cause statistics.

### Multicenter study

#### Study population

Children aged > 27 days and < 18 years admitted to a children’s hospital in NRW during the study period due to a community-acquired infection with *S. pyogenes*, *S. pneumoniae*, *N. meningitidis*, or *H. influenzae* were eligible, including invasive and non-invasive infections but no catheter-associated or nosocomial infections.

#### Clinical data collection

Eligible patients were identified via positive test results for the pathogen of interest by the local microbiology departments or derived from diagnose related group codes (International Classification of Diseases, 10th revision, German modification, ICD-10-GM). Eligibility was confirmed via retrospective chart review by the local investigators and de-identified clinical data entered into web-based case report forms at www.limesurvey.org.

Central nervous system (CNS) infections were defined as infections limited to the CNS (e.g., cerebrospinal fluid, intracranial abscess/empyema). Eye and ear–nose–throat (E+ENT) infections were defined as infections primarily located in the upper respiratory tract down to the larynx, including paranasal sinuses, Eustachian tubes, middle ear, mastoid cavities, and orbita. E+ENT with cerebral invasion were defined as primary E+ENT infections with penetration into the CNS.

Concomitant viral infections were diagnosed by the local laboratories via swabs from the respiratory tract by polymerase chain reaction or antigen testing. No information was available on viral infections preceding admission and vaccination status.

In cases with detection of multiple pathogens or non-invasive infection, the relevant pathogen was determined by a pediatric infectious disease specialist (S.G.) and a pediatric intensivist with additional qualification as infectious disease specialist (C.D.S.) according to clinical and laboratory findings. Pneumonia was confirmed either from blood culture, pleural empyema or tracheal secretion/bronchoalveolar lavage fluid. In cases with adequate antibiotic treatment prior to hospitalization, pneumonia was also accepted if radiologic signs were present and another mode of detection was positive for the respective pathogen, e.g., pneumococcal antigen in urine or *S. pyogenes* antigen in throat swabs.

Duplicate reports in the dataset originating from inter-center referrals were removed after updating the total duration of hospital stay.

#### Children’s hospital capacities of NRW

Each participating hospital reported the number of non-surgical beds on pediatric wards (including neonatal and pediatric intensive care units (ICU)). Surgical beds that are not strictly designated for surgical patients but can be used for either surgical or non-surgical patients as needed were counted as pediatric. The capacities of the three non-participating centers were obtained by inquiry to the respective children’s hospital.

### Surveillance of invasive bacterial infections

Reporting of invasive infections by the studied pathogens is mandatory except for *S. pyogenes* (Supplementary Table S1). Incidences used in this study are from NRW cases only. Serotypes of *S. pneumoniae* and *H. influenzae* and serogroups of *N. meningitidis* were provided by the nationwide reference laboratories (NRL) to assess vaccine-preventability of invasive infections in two time periods: 2016–2021 and 2022–03/2023.

### Population and death cause statistics

Nationwide and NRW mid-year populations for cases < 18 years of age from 2016 to 2022 were derived from the Federal Statistical Office (FSO). Annual deaths from streptococci, *N. meningitidis*, and *H. influenzae* infections from 2016 to 2021 were extracted via ICD-10-GM codes from national death cause statistics provided by the FSO (Supplementary Table S2). Because age grouping at the FSO is available in 5-year categories, expected deaths were calculated only for children < 15 years of age. The number of expected deaths per year in NRW was extrapolated from the proportion of children living in NRW compared to the total pediatric population of Germany (22%).

### Linkage of clinical and surveillance data

Clinical MC and NRL surveillance data of *S. pyogenes*, *S. pneumoniae, N. meningitidis* and *H. influenzae w*ere matched by patient’s age, sex, sample collection site and date, identified pathogen, and serotype if applicable (full agreement of all items was considered a match). Direct record linkage by name or date of birth was inapplicable due to de-identification and prohibited by general data protection regulations.

### Statistical analyses

Continuous variables are presented as means with 95% confidence intervals (CI) if evenly distributed and as median with interquartile range (IQR) if skewed. Discrete variables are presented as counts and percent. The dataset of the MC study contained no missing data.

Capture–recapture analyses (CRC) were conducted using a generalized log-linear model. CRC analysis is a statistical method used to estimate the size of a population by capturing, marking, and releasing individuals, and then recapturing a portion of them later. It involves comparing the marked and unmarked individuals in the recaptured sample to make inferences about the total population size. In this study, we matched the cases from MC study and the cases reported to the NRLs to identify the overlap (= recaptured cases) and estimate the total number of severe infections for each respective pathogen.

Monthly overall and pathogen-specific incidence rates per 100.000 child years (CY) were calculated from total case numbers estimated by CRC analyses assuming a consistent reporting rate over time. Monthly incidence rate ratios (IRR) were calculated for the period from January 2020 to March 2023, with the corresponding months of 2016–2019 as references. Besides pathogen-specific incidence rates and IRRs, cumulative monthly incidence rates and IRRs were calculated.

The expected number of deaths from the studied pathogens in cases < 15 years of age was calculated from the average number of annual deaths in Germany and the fraction of children living in NRW.

SAS Enterprise Guide 8.3 (SAS Institute Inc., Cary, NC, USA) was used to perform statistical analyses and produce figures.

### Ethics approval

The study was approved by the Ethics Committee of the Medical Faculty of the University of Duisburg-Essen (22-11045-BO).

## Results

Fifty-nine of 62 (95%) children’s hospitals in NRW participated in the MC study, comprising 4066 of 4323 (94%) pediatric hospital beds. At the end of 2022, 22% of Germany’s children lived in NRW. Subtracting the 6% capacities from non-participating hospitals, the participating centers supplied hospital care for approximately 20.7% of German children.

**Fig. 1:**
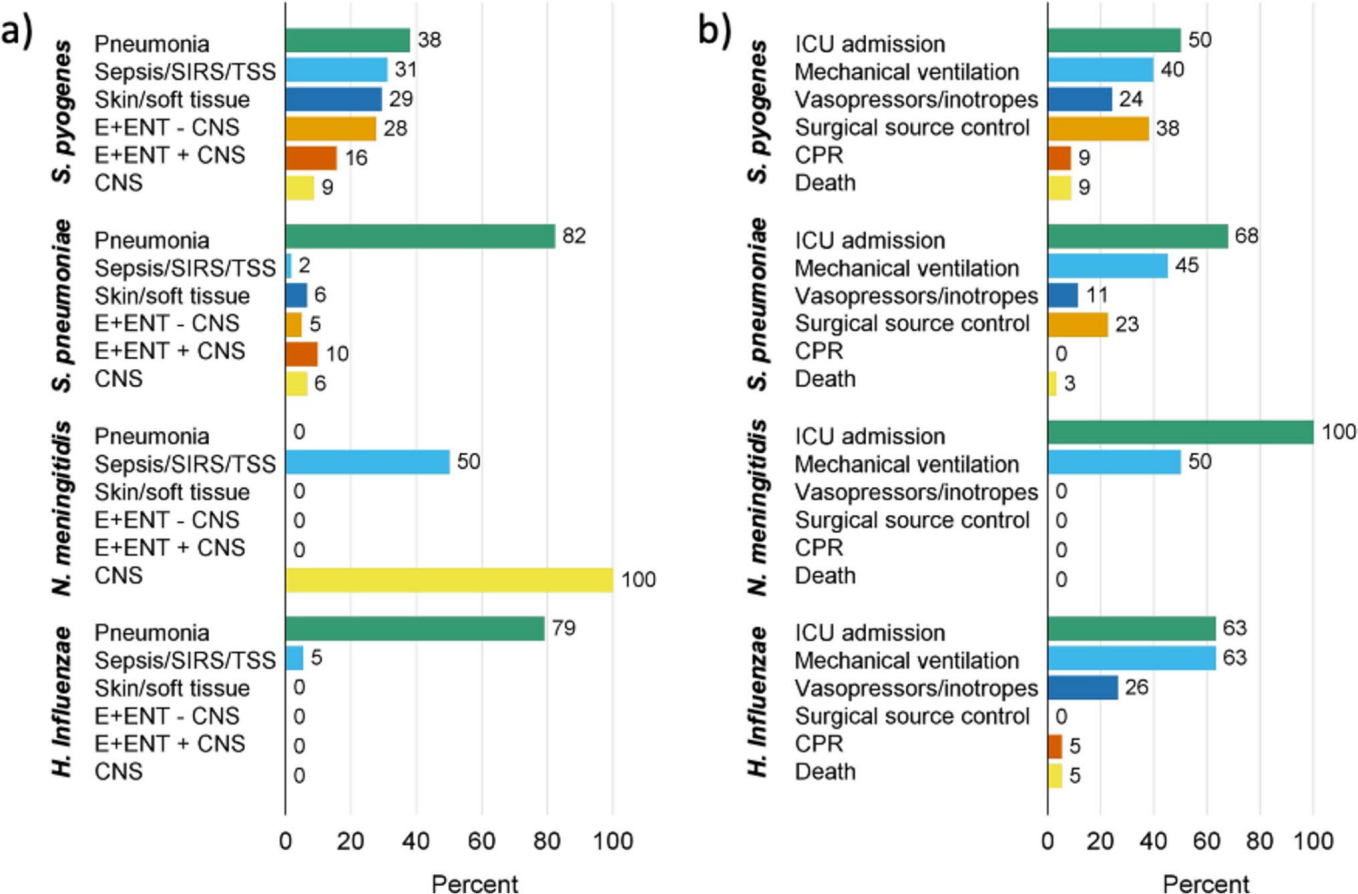
Hospital-treated community-acquired bacterial infections in children in North Rhine-Westphalia in the last quarter of 2022. **a** Clinical presentation. **b** Treatment and outcomes. *SIRS* systemic inflammatory response syndrome, *TSS* toxic shock syndrome, *ENT* ear–nose–throat, *CNS* central nervous system, *ICU* intensive care unit, *CPR* cardiopulmonary resuscitation

A total of 153 cases were reported with a median age of 4 years (IQR 1–7). Fifty-eight (38%) cases were from *S. pyogenes*, 62 (41%) from *S. pneumoniae*, 2 (1%) from *N. meningitidis*, and 19 (12%) from *H. influenzae* (Table 1). The most frequent presentation was pneumonia (*n* = 91, 59%), followed by sepsis/systemic inflammatory response syndrome/toxic shock syndrome (*n* = 28, 18%), skin or soft tissue infections (*n* = 22, 14%), and E+ENT infection without cerebral invasion (*n* = 22, 14%). Viral co-infections were observed in 71 (46%) cases. ICU admissions were frequent (*n* = 90, 59%), as was mechanical ventilation (n = 67, 44%), surgical source control (*n* = 41, 27%), and use of vasopressors or inotropes (*n* = 26, 17%) (Table 1, Fig. 1). Eight children died (5%), and five (3%) were discharged to a rehabilitation or care facility. Overall functional neurological outcome at hospital discharge in survivors was good (median pediatric cerebral performance category (PCPC) = 1, IQR 1–2). The sites of pathogen detection in the MC study and the proportion of invasive infections varied between the pathogens, with the highest absolute burden of invasive infections caused by *S. pyogenes* and *S. pneumoniae *(Table 2).

**Table 1:** Clinical characteristics of hospital admissions due to bacterial infections reported in a multicenter study in children’s hospitals in North Rhine-Westphalia (Germany) between October 1st and December 31st, 2022

	Total	*S. pyogenes*	*S. pneumoniae*	*H. influenzae*	*N. meningitidis*	*S. *spp.^a^
	*N* = 153 (100%)	*n* = 58 (38%)	*n* = 62 (41%)	*n* = 19 (12%)	*n* = 2 (1%)	*n* = 12 (8%)
Male, *n *(%)	86 (56%)	39 (67%)	30 (48%)	10 (53%)	1 (50%)	6 (50%)
Age in years, *median (IQR)*	4 (1–7)	4 (2–7)	2.5 (1–5)	3 (0–6)	9.5 (2–17)	3.5 (0.5–8.5)
Viral co-infection, *n *(%)	71 (46%)	18 (31%)	37 (60%)	14 (74%)	0 (0%)	2 (17%)
RSV, *n *(%)	21 (14%)	2 (3%)	10 (16%)	8 (41%)	0 (0%)	1 (8%)
Influenza A/B, *n *(%)	29 (19%)	12 (21%)	14 (23%)	3 (16%)	0 (0%)	0 (0%)
SARS-CoV-2, *n *(%)	3 (2%)	1 (2%)	2 (3%)	0 (0%)	0 (0%)	0 (0%)
Other, *n *(%)	24 (16%)	4 (7%)	17 (27%)	3 (16%)	0 (0%)	1 (8%)
Bacterial co-infection with *H. influenzae*, *n *(%)	12 (8%)	1 (2%)	10 (16%)	-	0 (0%)	1 (8%)
Clinical presentation						
Sepsis, SIRS, Toxic Shock, *n *(%)	28 (18%)	18 (31%)	1 (2%)	1 (5%)	1 (50%)	7 (58%)
E + ENT infections w/o cerebral invasion, *n *(%)	22 (14%)	16 (28%)	3 (5%)	2 (11%)	0 (0%)	3 (25%)
E + ENT infections w/ cerebral invasion, *n *(%)	15 (5%)	9 (2%)	6 (10%)	0 (0%)	0 (0%)	0 (0%)
CNS infection, *n *(%)	15 (10%)	5 (9%)	4 (7%)	0 (0%)	2 (100%)	4 (33%)
Pneumonia, *n *(%)	91 (59%)	22 (38%)	51 (82%)	15 (79%)	0 (0%)	3 (25%)
With pleural empyema	37 (24%)	16 (28%)	19 (31%)	2 (11%)	0 (0%)	2 (17%)
Skin and soft tissue infections, *n *(%)	22 (14%)	17 (29%)	4 (7%)	0 (0%)	0 (0%)	1 (8%)
Other infections, *n *(%)	5 (3%)	2 (3%)	0 (0%)	1 (5%)	0 (0%)	3 (25%)
Treatment						
Resuscitation, *n *(%)	6 (4%)	5 (9%)	0 (0%)	1 (5%)	0 (0%)	0 (0%)
PICU admission, *n *(%)	90 (59%)	29 (50%)	42 (68%)	12 (63%)	2 (100%)	4 (33%)
Invasive ventilation, *n *(%)	67 (44%)	23 (40%)	28 (45%)	12 (63%)	1 (50%)	3 (25%)
Administration of vasopressors/inotropes, *n *(%)	26 (17%)	14 (24%)	7 (11%)	5 (26%)	0 (0%)	0 (0%)
Extracorporeal membrane oxygenation, *n *(%)	1 (1%)	1 (2%)	0 (0%)	0 (0%)	0 (0%)	0 (0%)
Surgical source control, *n *(%)	41 (27%)	22 (38%)	14 (23%)	0 (0%)	0 (0%)	7 (58%)
Length of hospital stay hospital (days), median (IQR)	10 (5–19)	8 (4–19)	11 (5–19)	5 (4–6)	6 (1–11)	13 (7.5–25.5)
Discharge to rehabilitation/care facility, *n *(%)	5 (3%)	2 (3%)	2 (3%)	0 (0%)	0 (0%)	1 (8%)
PCPC at hospital discharge, median (IQR)	1 (1–2)	1 (1–2)	1 (1–2)	1 (1–2)	1 (1–1)	1 (1–2)
Deceased, *n *(%)	8 (5%)	5 (9%)	2 (3%)	1 (5%)	0 (0%)	0 (0%)

*IQR* interquartile range, *ENT* ears nose and throat, *CNS* central nervous system, *SIRS* systemic inflammatory response syndrome, *PICU* pediatric intensive care unit, *PCPC* Pediatric Cerebral Performance Category

^a^Streptococcal species other than *S. pyogenes* and *S. pneumoniae*

**Table 2:** Sites of pathogen detection in the multicenter study

Site of pathogen detection	*S. pyogenes* (*n* = 58)	*S. pneumoniae* (*n* = 62)	*H. influenza* (*n* = 19)	*N. meningitides* (*n* = 2)
Sterile sites				
Blood culture	24 (41%)	8 (13%)	4 (21%)	1 (50%)
Cerebrospinal fluid culture	2 (3%)	4 (6%)	0 (0%)	1 (50%)
Bronchoalveolar lavage/tracheal secretion	2 (3%)	14 (23%)	6 (32%)	0 (0%)
Pleural empyema	16 (28%)	14 (23%)	2 (11%)	0 (0%)
Intraoperative swab	9 (16%)	3 (5%)	0 (0%)	0 (0%)
Synovial fluid	2 (3%)	0 (0%)	0 (0%)	0 (0%)
Abscess	2 (3%)	0 (0%)	0 (0%)	0 (0%)
Non-sterile sites				
Sputum/throat swab	12 (21%)	25 (40%)	11 (58%)	0 (0%)
Skin/soft tissue/wound swab	6 (10%)	0 (0%)	1 (5%)	0 (0%)
Antigen rapid test	3 (5%)	0 (0%)	0 (0%)	0 (0%)
Antigen test (urine)	0 (0%)	7 (11%)	0 (0%)	0 (0%)
Other	2 (3%)	0 (0%)	0 (0%)	0 (0%
Invasive infection according to NRL definition	43 (74%)	36 (58%)	7 (37%)	2 (100%)

*NRL* nationwide reference laboratory

Among NRL-reported cases of invasive infections, the degree of vaccine-preventability varied depending on the pathogen and changed over time (Table 3). In *S. pneumoniae*, the proportion of vaccine-preventable cases increased, whereas they decreased in *H. influenzae and N. meningitidis.*

**Table 3:** Serotypes/serogroups of *S. pneumoniae, H. influenzae,* and *N. meningitidis* registered with the Nationwide Reference Laboratories in children in North Rhine-Westphalia (Germany), 2016–2023

Pathogen		2016–2021	2022–03/2023
*S. pneumoniae*	Serotype	*n* = 235	*n* = 75
	Preventable with PCV13*^a^	56 (24%)	28 (37%)
	Preventable with PCV15*^b^	65 (28%)	29 (39%)
	Preventable with PCV20*^c^	123 (52%)	33 (44%)
	Preventable with PCV23*^d^	134 (57%)**	38 (51%)
	Non-vaccine-preventable	100 (43%)	37 (49%)
*H. influenzae*	Serotype	*n* = 62	*n* = 25
	b	17 (27%)	2 (8%)
	Non-vaccine preventable	45 (73%)	23 (92%)
*N. meningitidis*	Serogroup	*n* = 98	*n* = 18
	B	79 (81%)	13 (72%)
	C	3 (3%)	1 (6%)
	W	4 (4%)	0 (0%)
	X	0 (0%)	1 (6%)
	Y	12 (12%)	3 (17%)
	Serogroup missing	1 (1%)	0 (0%)

^*^^a^PCV13 serotypes: 1, 3, 4, 5, 6A, 6B, 7F, 9 V, 14, 18C, 19A, 19F, 23F

^*^^b^PCV15 serotypes: 1, 3, 4, 5, 6A, 6B, 7F, 9 V, 14, 18C, 19A, 19F, 22F, 23F, 33F

^*^^c^PCV20 serotypes: 1, 3, 4, 5, 6A, 6B, 7F, 9 V, 10A, 11A, 12F, 14, 15B, 18C, 19A, 19F, 22F, 23F, 33F

^*^^d^PCV23 serotypes: 1, 2, 3, 4, 5, 6B, 7F, 8, 9N, 9 V, 10A, 11A, 12F, 14, 15B, 17F, 18C, 19A, 19F, 20, 22F, 23F, 33F

^**^One case with serotype 6A was preventable with PCV13, PCV15 or PCV20 but not with PCV23

CRC analyses showed different degrees of overlap between the MC study and NRL data. For *N. meningitidis*, capture by the NRL was complete, but the total incidence (*n* = 6, sporadic infections—no outbreak) was low compared to the estimated total numbers of *S. pneumoniae* (*n*_estimated_ = 232), *S. pyogenes* (*n*_estimated_ = 214), and *H. influenzae* (*n*_estimated_ = 95) (Table 4).

**Table 4:** National surveillance and multicenter study detection of pediatric severe and invasive bacterial infections between October 1st and December 31st of 2022 in North-Rhine Westphalia (Germany)

Pathogen	Captured by			Estimated number of non-captured cases	Estimated total number of cases
	NRL only	MC only	Overlap of NRL and MC		
	*N*	*n*	*n*	*n* (95% CI)	*n*
*S. pyogenes*	18	58	5	138 (48–400)	214
*S. pneumoniae*	18	63	5	151 (52–437)	232
*N. meningitidis*	6	2	2	0 (0–0)^a^	6
*H. influenzae*	6	31^b^	2	58 (10–329)	95

*NRL* nationwide reference laboratory, *MC* multicenter study

^a^Exact estimate: 2.1E-11 (9.4E-12–4.6E-11)

^b^Primary and co-infections included

**Fig. 2:**
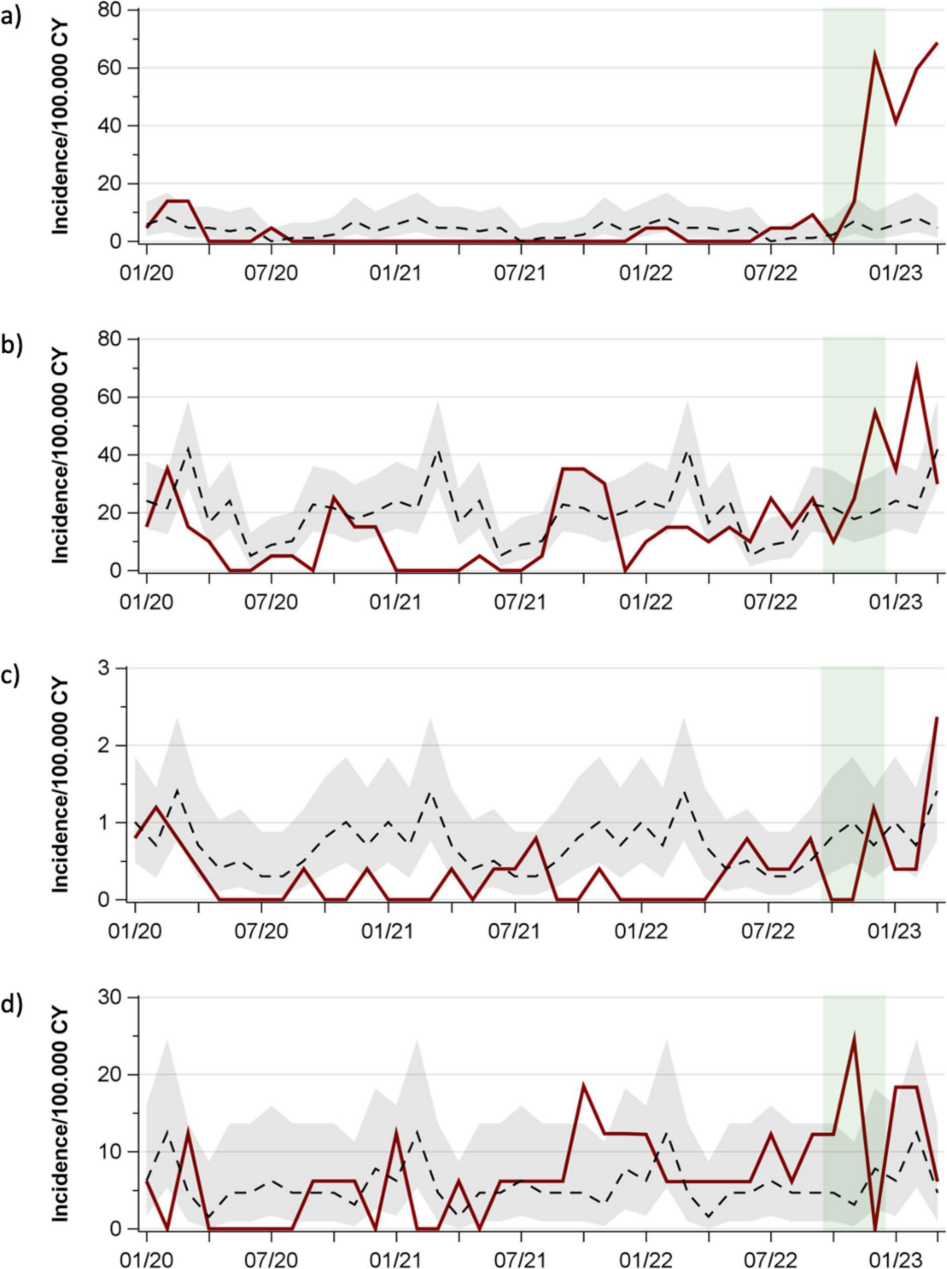
Hospitalizations for community-acquired bacterial infections. **a** *S. pyogenes*. **b** *S. pneumoniae*. **c** *N. meningitidis*. **d** *H. influenzae*. Red solid line: incidence rates January 2020–March 2023; black dashed line and grey band: average monthly incidence rates 2016–2019 with 95% CI; green band: period of MC study

**Fig. 3:**
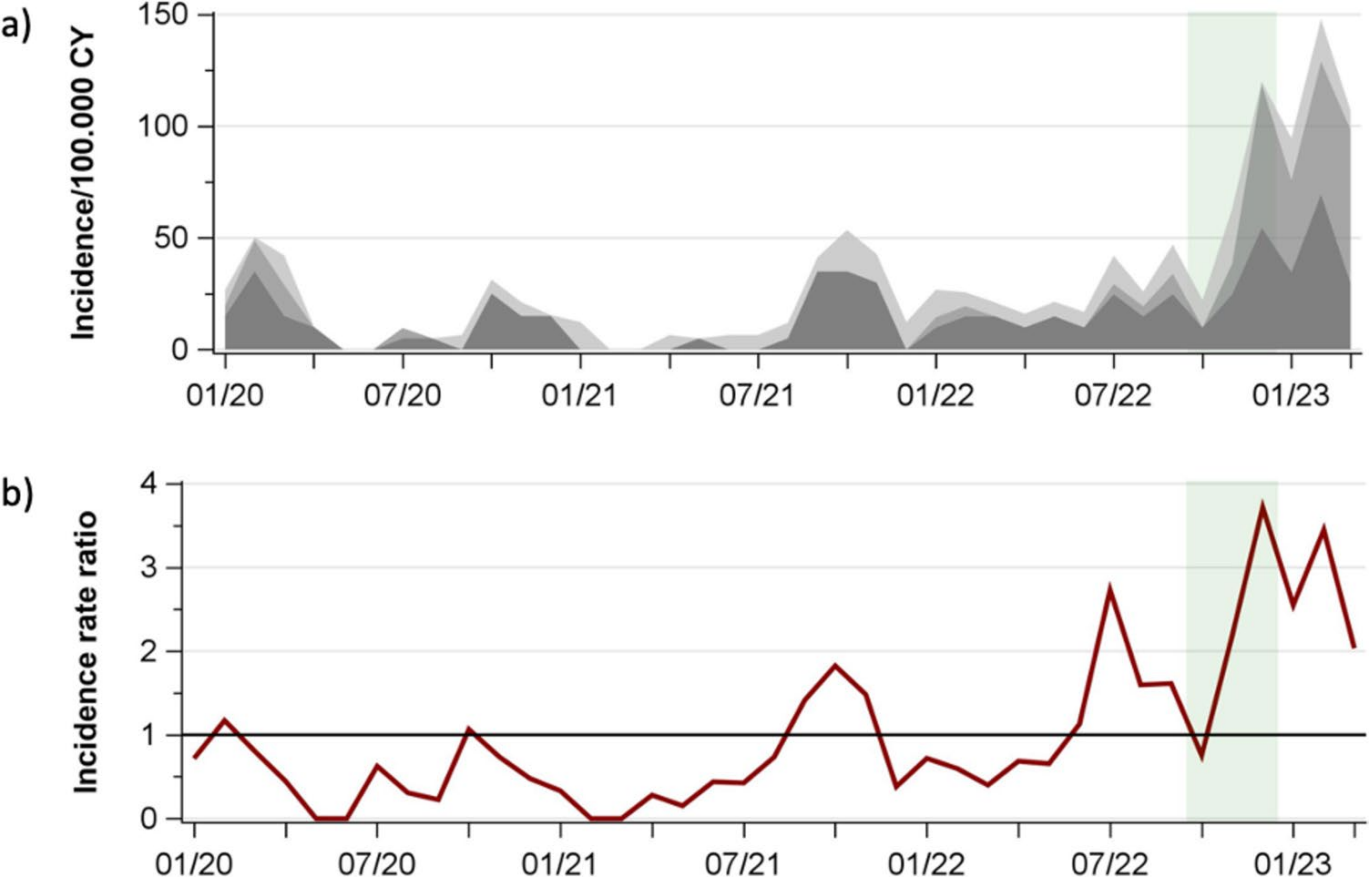
Hospitalizations due to acquired-bacterial infections from S. pyogenes, S. pneumoniae, *N. meningitidis*, and *H. influenzae*, January 2020–March 2023. **a** Cumulative hospitalization rates of S. pneumoniae (dark grey), S. pyogenes (medium grey), and *N. meningitidis* + *H. influenzae* (light grey). **b** IRRs of infections, reference period 2016–2019. Green band: period of MC study

With onset of the COVID-19 pandemic, incidence rates and IRRs exhibited a decline, followed by a pathogen-specific degree of recovery up to or above pre-pandemic levels (Figs. 2 and S1 and Supplementary Table S2). Alike, cumulative monthly incidence rates and IRRs declined at the beginning of the pandemic, showed small peaks during the course of the pandemic, and peaked in December 2022 and February 2023 (Fig. 3 and Supplementary Tables S2 and S3).

The reported number of deaths in children < 15 years in the MC study exceeded the expected number of annual deaths from *S. pyogenes* and *S. pneumoniae* (7 observed; 0.9 expected). No deaths were caused by *N. meningitidis* (1.8 expected) and *H. influenzae* (0 expected) (Table 5). The death from *H. influenzae* in the MC study occurred in a patient aged 17 years and is, therefore, not included in this statistic.

**Table 5:** Expected and observed number of deaths in children < 15 years in Germany categorized by pathogen

Pathogen	Expected number of deaths in Germany per year^a^	Expected number of deaths in NRW per year^a^	Observed number of deaths in multicenter study in last quarter of 2022
Streptococci (all species)	4	0.9	7
*N. meningitidis*	8	1.8	0
*H. influenzae*	0.3	0.1	0^b^

^a^Based on national death cause statistics 2016–2019

^b^One death from *H. influenzae *occurred in a patient aged 17 years and is, therefore, not included in the table

## Discussion

This multi-source study in Germany’s most populous federal state detected high incidences of pediatric general ward and pediatric ICU admissions for bacterial infections associated with *S. pyogenes, S. pneumoniae*, *N. meningitidis*, and *H. influenzae* in the last quarter of 2022. Disease severity was high with more than half of the cases admitted to an ICU and frequent requirement of invasive mechanical ventilation and vasoactive agents. Longitudinal assessment since 2016 revealed an unprecedented peak of bacterial infection-related hospitalizations in late 2022 and early 2023, primarily driven by *S. pyogenes* and *S. pneumoniae*. Deaths during the 3-month MC study period were seven times higher than expected for the entire year.

A major finding that has already been reported in adults and children [14, 15] was the marked overall decline of severe and invasive bacterial infections at the beginning of the COVID-19 pandemic. In our study, this overall decline was shortly interrupted in fall 2021 (when schools and day care facilities re-opened) but otherwise lasted until early summer 2022, coinciding with the timepoint when compulsory wearing of protective face masks was abrogated.

A decline of *S. pyogenes* infections from the onset of the pandemic until spring 2022 was also reported in France, followed by a sudden increase of non-invasive *S. pyogenes* infections [16]. Like Germany, France and England also witnessed strong increases of severe and invasive *S. pyogenes* infections during fall and winter of 2022 [7, 17]. These surges were most likely induced by an overall rise in infection numbers with the proportion of severe cases remaining constant, rather than changes in types, virulence or toxicity [7, 18–20]. A possible explanation is the discontinuation of face mask wearing among children, which re-enabled droplet transmission of *S. pyogenes*, an essential mechanism of infection [21]. High prevalence of acute respiratory virus infections as reported by the Robert Koch-Institute at the height of the *S. pyogenes* wave [22] may have paved the way for bacterial superinfections. Superinfections are described for influenza virus in combination with *S. pyogenes* and *S. pneumoniae* [23] and were also observed in our study.

Our study found viral co-infections in 60% of the *S. pneumoniae* cases, most commonly by influenza and respiratory syncytial virus. In line with this finding, asymptomatic *S. pneumoniae* carriage and respiratory virus infection as starting point for invasive pneumococcal disease is well-acknowledged [24]. During the pandemic, the decline in pneumococcal infections in Germany was less prominent compared to *S. pyogenes* infections, possibly due to viral infection waves among children in spite of pandemic measures [22]. In contrast to *S. pyogenes* infections, invasive pneumococcal disease can be effectively prevented by vaccination [25], necessitating high levels of vaccination rates to attenuate virus-triggered outbreaks. We were not able to assess the vaccination status of the included cases in the MC study but the cases registered by the NRL show an increase of invasive pneumococcal infections by vaccine-preventable serotypes. This finding aligns with reports that the German population is incompletely vaccinated against pneumococci: Pneumococcal vaccination coverage for children entering primary school increased from 15% in 2010 to 83% in 2017–2019 but is only 2% in adolescents and adults [26, 27]. Of infants born in 2016, only 73% received a full series of the recommended pneumococcal vaccination scheme and vaccinations were frequently delayed [28]. This low vaccination coverage and the observed increase of invasive infections from the serotypes recommended for basic immunization in infants by the German Standing Committee on Vaccination (STIKO) calls for urgent improvement to enhance protection against opportunistic pneumococcal infections. Additionally, vaccine coverage against influenza, SARS-CoV-2, and respiratory syncytial virus should be optimized to reduce the susceptibility to bacterial and superinfections.

Vaccines are also responsible for decreasing incidences of meningococcal infections in several countries [29]. In Germany, vaccination is recommended for serogroup C with a subsequent decline of serogroup C infections since its introduction [30]. The trend of decreasing meningococcal disease was further enhanced during the COVID-19 pandemic [14] and also noticeable in our study with incidence rates below pre-pandemic average. Yet, in March 2023, an increase in meningococcal infection incidence above average was observed but characterized by very low absolute numbers. In contrast to *S. pneumoniae*, vaccine coverage against meningococcal serogroup C in school starters in Germany is above 90% [27]. Alike, infections reported to the NRLMHi by serogroup C were rare. The majority was caused by serogroup B, which is also vaccine-preventable but not officially recommended by the STIKO and thus not covered by health insurances. Thus, coverage of serogroup B vaccination by health insurances represents a possible lever to reduce meningococcal infections in Germany even further.

For *H. influenzae*, a decline of invasive infections during the COVID-19 pandemic was observed in the Netherlands but associated with an increase of vaccine-preventable serotype b [31]. In our study, *H. influenzae* hospitalizations during the pandemic remained within seasonally expected ranges, showing only two peaks in December of 2021 and 2022, respectively. The NRLMHi recorded only 2 cases by serotype b, in line with the high vaccine coverage of 90% in German children against *H. influenzae b* [27]. Yet, IRRs remained almost constantly above one since June 2021, indicating a slight increase of non-vaccine *H. influenzae* infections, which contribute to the overall burden of pediatric hospitalizations.

Limitations of the study mainly derive from missing cases and information: Due to the incomplete coverage of children’s hospitals in NRW, the MC study missed 6% of pediatric hospital capacities. Cases treated outside of pediatric units, such as otorhinolaryngology or adult units, and pre-hospital deaths could not be captured, potentially leading to underestimation of incidences and disease severity. Variable pathogen-specific underreporting to the NRLs occurred, as commonly observed in infectious disease surveillance [32–34]. However, awareness of streptococcal infections due to reports from other countries may have increased reporting rates, causing overestimation of the incidence peak. Another limitation is the lack of information on viral infections preceding admission and vaccination status. Because it was not recorded if and which viral swabs patients received, we may have underestimated the proportion of viral co-infections.

In summary, the acute overload of the German pediatric health care system was caused by a rebound of acute viral and bacterial respiratory and invasive diseases that peaked at the same time, creating an unprecedented incidence of hospitalizations in children. Shortages of essential drugs, such as antibiotics and antipyretics, in the outpatient sector [35, 36] and a decrease in numbers of operable beds in children’s hospitals due to personnel shortages [37, 38] aggravated the pediatric health care crisis.

The results of this study indicate that unintended effects from preventive measures against the COVID-19 pandemic potentially continue to burden children’s health. While this is well-acknowledged for mental health and obesity, which directly increase morbidity but not mortality [39–42], the drastic increase of severe bacterial infections is new and its origin deserves deeper investigation. Further research should untangle this amalgam to quantify excess morbidity and mortality from acute bacterial infections themselves and the lack of adequate pediatric care capacities in Germany. However, even more urgent than scientific workup, is the implementation of effective measures against future outbreaks among children to prevent morbidity and deaths. These include high vaccination coverage and acquisition of natural immunity against both viral and bacterial disease, sufficient pediatric drug supply, and pediatric health care capacities adapted to seasonal demands.

### Supplementary Information

Below is the link to the electronic supplementary material.

Figure S1. Monthly incidence rate ratios of invasive bacterial infections in children in North Rhine-Westphalia January 2020–March 2023 (reference period 2016–2019). a) S. pyogenes. b) S. pneumoniae. c) N. meningitidis. d) H. influenzae

Supplementary table 3 (XLSX 23 KB). Excel file containing monthly NRW case numbers, incidence rates, and incidence rate ratios with 95 % confidence intervals from the national reference laboratories

Supplementary tables S1 and S2 (DOCX 42 KB).

## Data Availability

The data generated for this study are available without undue reservation to any qualified researcher upon reasonable request.
