# Supplementary figures and images for: Outbreak of severe community-acquired bacterial infections among children in North Rhine-Westphalia (Germany), October to December 2022
Source: Infection. 2024 Feb 16;52(3):1099–111. doi: 10.1007/s15010-023-02165-x (PMC11143032; doi:10.1007/s15010-023-02165-x)

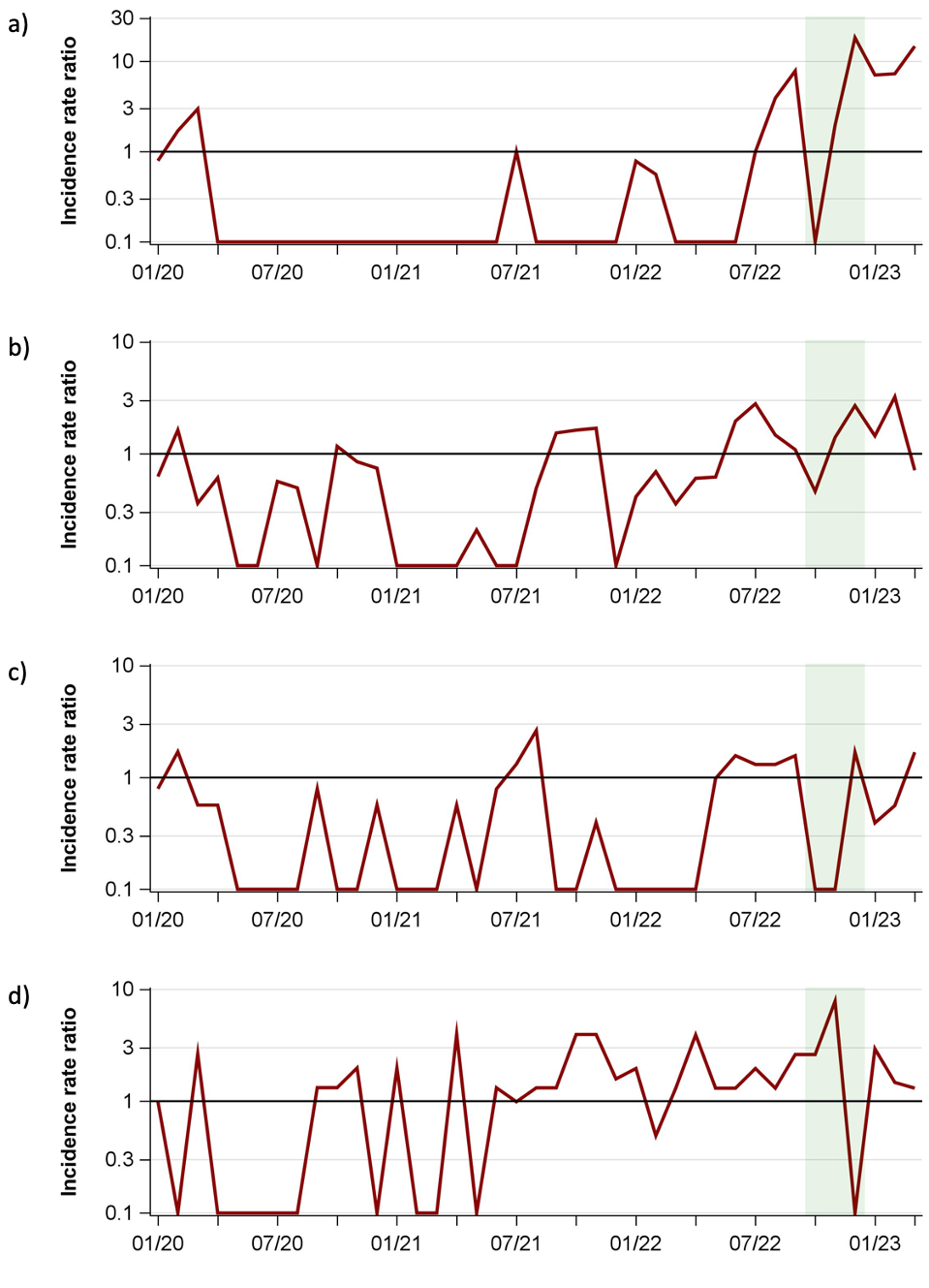

Supplement: Supplementary file 1 — Figure S1. Monthly incidence rate ratios of invasive bacterial infections in children in North Rhine-Westphalia January 2020–March 2023 (reference period 2016–2019). a) S. pyogenes. b) S. pneumoniae. c) N. meningitidis. d) H. influenzae [file 15010_2023_2165_MOESM1_ESM.png]
