# Supplementary material for: Outbreak of severe community-acquired bacterial infections among children in North Rhine-Westphalia (Germany), October to December 2022
Source: Infection. 2024 Feb 16;52(3):1099–111. doi: 10.1007/s15010-023-02165-x (PMC11143032; doi:10.1007/s15010-023-02165-x)
Supplement: Supplementary file 3 — Supplementary tables S1 and S2 (DOCX 42 KB). [file 15010_2023_2165_MOESM3_ESM.docx]

**Table S1:** Nationwide surveillance of invasive bacterial infections by streptococci, *N. meningitidis* and *H. influenzae* in Germany

| **Pathogen** | **Mode of surveillance** |
| --- | --- |
| Streptococci (all species) | Reporting of invasive S. pneumoniae infections is mandatory in Germany. However, surveillance of invasive streptococcal disease by the German reference laboratory for streptococci (GRLS) is based on voluntary reporting by primary diagnostic laboratories, which are quested to send all streptococcal isolates from invasive infections (blood, CSF or any otherwise normally sterile site) to the RLS for detailed analyses. This includes mainly microbiological, biochemical, serological and molecularbiological methods. |
| *N. meningitidis* and *H. influenzae* | These two pathogens are monitored in a joint institution, the National Reference Laboratory for *N. meningitidis* and *H. influenzae* (NRLMHi). Reporting of meningococcal meningitis and meningococcal septicaemia is mandatory in Germany. Additionally, the detection of *N. meningitidis* in blood, cerebrospinal fluid, petechiae, or other sterile sites and the detection of *H. influenzae* in blood or cerebrospinal fluid, must be reported. Submission of bacterial isolates or clinical specimens to the NRLMHi is voluntary. Up to 90% of the reported cases are covered by the submissions to NRLMHi. Following species confirmation, meningococcal serogroups and *H. influenzae* serotypes are determined. |

**Table S2:** International classification of diseases codes used to calculate the number of expected deaths from bacterial infections in children in Germany

| **Pathogen** | **ICD-10-GM code** |
| --- | --- |
| Streptococci (all species) | A40.0-A40.3, A40.8, A40.9, A46, A49.1, G00.1, G00.2, J03.0, J13, J15.3, J15.4, J20.2, M00.2, J02.0 |
| *N. meningitidis* | A39.0-A39.4, A39.8, A39.9 |
| *H. influenzae* | A41.3, A49.2, J14 |

ICD-10-GM = international classification of diseases 10 (German modification)
